# Oncogenic functions and clinical significances of DCLK1 isoforms in colorectal cancer: a systematic review and meta-analysis

**DOI:** 10.1186/s12935-022-02632-9

**Published:** 2022-06-18

**Authors:** Elham Kalantari, Mahdieh Razmi, Fatemeh Tajik, Mohsen Asadi-Lari, Roya Ghods, Zahra Madjd

**Affiliations:** 1 Oncopathology Research Center, Iran University of Medical Sciences (IUMS), Tehran, Iran; 2 Department of Epidemiology, School of Public Health, Iran University of Medical Sciences, Tehran, Iran; 3 Department of Molecular Medicine, Faculty of Advanced Technologies in Medicine, Iran University of Medical Sciences (IUMS), Tehran, Iran

**Keywords:** Colorectal cancer, DCLK1-S, DCLK1-L, Oncogenic functions, Meta-analysis

## Abstract

**Background:**

The oncogenic role of doublecortin-like kinase 1 (DCLK1) as a putative cancer stem cell (CSC) marker has been clarified in colorectal cancer (CRC). Isoform-specific functions of DCLK1 have shed new light on different functions of DCLK1 short (DCLK1-S) and DCLK1 long (DCLK1-L) isoforms in tumor initiation, growth, and metastasis. Therefore, the current systematic review and meta-analysis aimed to review the available in vitro, in vivo, and clinical evidence on the oncogenic roles and clinical significance of DCLK1 isoforms in colorectal cancer.

**Methods:**

The literature databases of PubMed, Scopus, ISI Web of Science, and Embase were searched to identify eligible articles. The description characteristics of in vitro and pre-clinical studies were extracted from identified reports. In addition, hazard ratios (HRs) or odds ratios (ORs) with 95% confidence intervals (CIs) were recorded to determine the relationships between DCLK1-L and DCLK1-S expression and prognostic outcomes in patients with CRC.

**Results:**

Both in vitro and in vivo evidence have emphasized the potential oncogenic functions of DCLK1 in tumor initiation, self-renewal ability, tumor invasion, epithelial-mesenchymal transition (EMT), and metastasis. However, the anti-DCLK1 antibodies generally utilized in these studies could detect sequence homology epitopes of both isoforms. Recent limited isoform-specific evidence has strongly supported the significant positive expression and rather oncogenic efficacy of DCLK1-S in tumorigenesis, EMT, and invasion compared with DCLK1-L in human CRC cell lines. Our meta-analysis findings of limited clinical studies indicated that only overexpression of DCLK1-S is associated with worse overall survival (OS) (HR = 7.930, 95% CI 2.252–27.924, *p* = 0.001). Increased expression of both DCLK1-S (HR = 1.610, 95% CI 1.020–2.541, *p* = 0.041) and DCLK1-L (HR = 5.890, 95% CI 1.219–28.453, *p* = 0.027) isoforms was closely associated with worse DSS/CSS in CRC patients. Furthermore, the high expression of DCLK1-S was found to be associated with poor DFS/RFS/PFS (HR = 1.913, 95% CI 1.230–2.973, *p* = 0.004).

**Conclusions:**

The current findings strongly supported that the DCLK1-S isoform may play a crucial role in the invasion, aggressive tumor behavior, and worsened survival outcomes of CRC patients. However, further critical investigations related to the potential preclinical and clinical utilities of DCLK1-S as a specific CRC-CSC marker are warranted.

**Supplementary Information:**

The online version contains supplementary material available at 10.1186/s12935-022-02632-9.

## Introduction

Colorectal cancer (CRC) is considered one of the leading causes of cancer mortality and counts as a major global public health concern [1]. Despite significant clinical and scientific improvements that have improved anti-cancer therapy, tumor development, metastasis, and recurrence have remained difficult outcomes in CRC patients. Identifying appropriate prognostic and diagnostic biomarkers represents a valuable promising tool to detect the disease in the early stage, predict clinical outcome and treatment failure, and reduce the mortality rate of patients with CRC. With the novel treatment approach of specifically targeting cancer stem cells, normal cells are spared. CSCs are a specific cancer cell subpopulation that has stem-like features and is responsible for tumor maintenance, development, and resistance to cancer therapy [2–4]. As a consequence, finding of specific CSC biomarkers, including Leucine-rich repeat-containing G-proteine-coupled receptor 5 (Lgr5), Nanog, Oct 4, CD166, and Aldehyde dehydrogenase isoform 1 (ALDH1), may shed new light on targeted-therapy strategies and lead to prolonged survival of patients with cancer [5, 6].

**Fig. 1:**
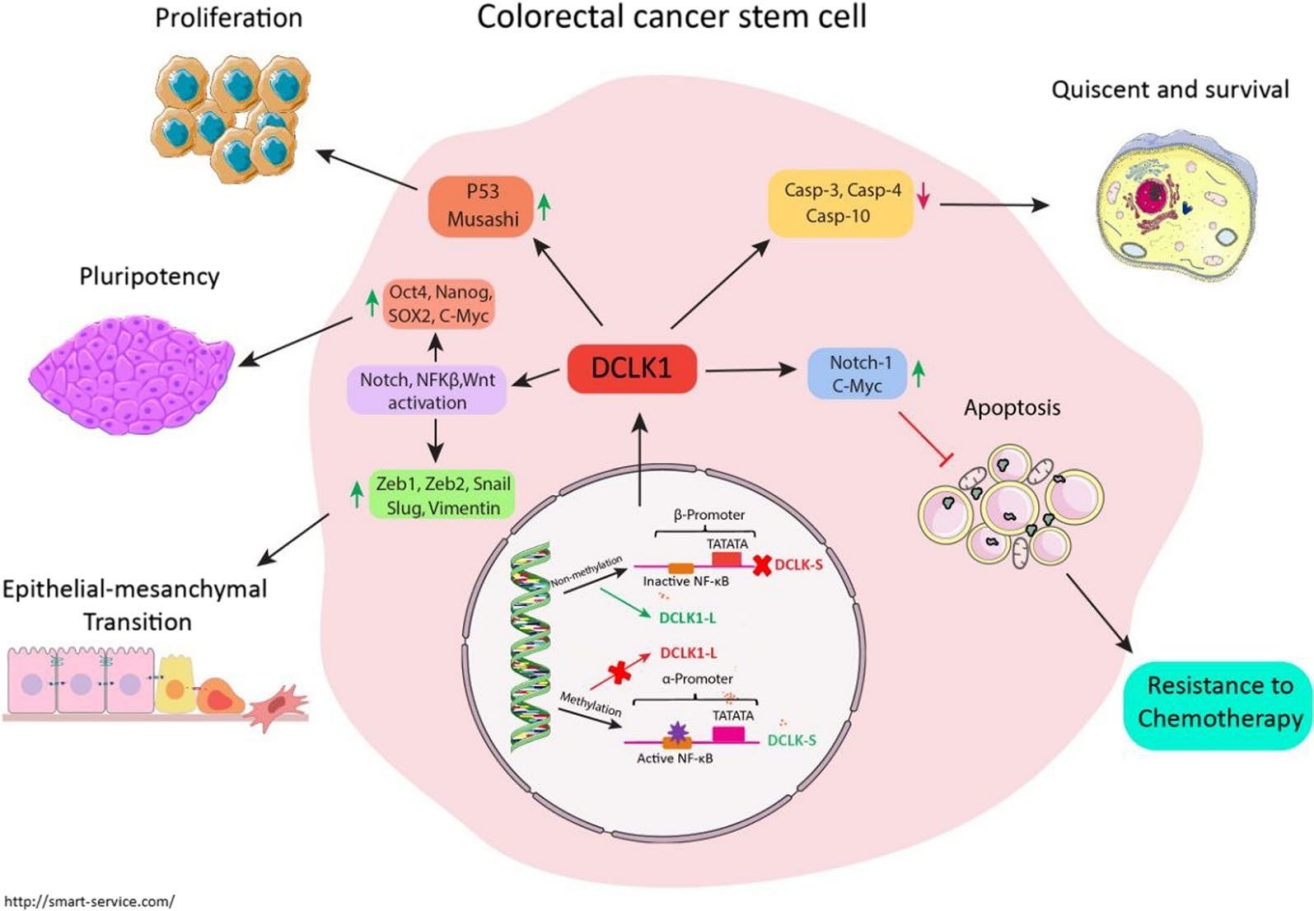
Oncogenic functions and carcinogenesis pathways of DCLK1 isoforms

Much of the initial research published by our and other groups have recently revealed the doublecortin-like kinase 1 (DCLK1) antigens as valuable predictive biomarkers and suitable candidates for tumor immunotherapy in terms of their role in regulating diverse tumorigenesis pathways [7–10]. The discovery of DCLK1 potentials to distinguish colorectal CSCs from normal stem cells in CRC highlighted DCLK1 as a colon cancer-specific marker [11]. DCLK1 isolation, identification, and targeting as an oncogenic driver indicates that DCLK1 may promote tumor heterogeneity and metastatic spread in colon and pancreas carcinomas [12–15]. Several studies supported the critical oncogenic role of DCLK1 CSC surface marker in the gastrointestinal (GI), colon, pancreases, and renal cell carcinomas (RCC) [16–23]. Preclinical studies have proved the biological functions of DCLK1 as a requisite factor in proliferative potential, angiogenesis, epithelial-mesenchymal transition (EMT), tumor invasion, and metastasis in solid tumors particularly in CRC (Fig. 1) [13, 22–26]. Growing evidence supports the regulatory role of DCLK1 in NOTCH, NFKB, and WNT molecular signaling pathways, emphasizing its contribution to carcinogenesis [16]. In RCC, the knocking down of DCLK1 via siDCLK1 transfection and its significant association with expression of EMT transcription factors SNAI1, SNAI2, TWIST1, ZEB1, and mesenchymal marker Vimentin protein demonstrated that inhibition of DCLK1 reduced the invasive and metastatic potential [23]. Sureban et al. reported a significant reduction in the expression of stem cell pluripotency factors MYC, NANOG, POU5F1/OCT4, and SOX2 by DCLK1 knocking down of DCLK1 in pancreatic cancer [27]. Several studies have linked DCLK1 overexpression to clinicopathological characteristics and poor prognosis in CRC patients, indicating that it may play a role in tumor development [14, 15]. Our previous investigations, in line with others, also indicated the significant association between higher expression of DCLK1 and aggressive nature of the tumor, advanced stages, and higher tumor grade on both protein and mRNA levels in CRC patients [8, 19, 28].

Recently, two distinct DCLK1 isoforms, including DCLK1 long isoform (DCLK1-L) (NM 004734.4) with 80–82 kDa, originated from 5'(α)-promoter and DCLK1 short isoform (DCLK1-S) (NM 001195415.1) with 45–50 kDa originated from an alternative transcriptional β-promoter have identified, that are characterized by different biological functions in human normal and cancerous brain and colon cells [12, 29, 30]. More than 98 percent similarity between DCLK1-L and DCLK1-S isoforms was observed at the C-terminal end of both proteins, with only a six-amino-acid difference found at the N-terminal end of the DCLK1-S protein [30]. Since commercial antibodies target the C-terminal domain of DCLK1, the unique oncogenic functions of DCLK1 isoforms, as well as their possible contribution to tumor growth in cancer tissues like CRC, need to be investigated further. Recently, Sarkar et al. reported the higher expression of DCLK1-S compared to DCLK1-L in patients and cell lines with CRC versus normal samples by generating specific anti-DCLK1-S antibodies [12, 30]. Moreover, increased expression of DCLK1-S was observed in high-risk CRC patients with shorter overall survival at the time of the colonoscopy [30]. Meanwhile, further comprehensive studies are required to find a definite conclusion about the biological implications of each isoform.

To date, some reviews were conducted regarding DCLK1 importance in cancer progression; however, data of the available literature do not provide a complete and systematic overview. Therefore, the present systematic review, for the first time, was conducted to review the available in vitro, in vivo, and clinical evidence on the oncogenic roles and clinical significance of DCLK1 isoforms in colorectal cancer, with the goal to highlight their clinical implications and develop novel DCLK1-targeted therapy possibilities.

## Methods

### Protocol and registration

This systematic review is registered in the International Prospective Register of Systematic Reviews (CRD42021273626). The review is reported according to the Preferred Reporting Items for Systematic Reviews and Meta-Analyses (PRISMA) statement [31].

### Search strategy

PubMed/MEDLINE, Scopus, Embase (Embase.com), and Web of Science were used as databases to search the relationship of DCLK1 expression with its oncogenic function (in vivo and in vitro), clinicopathological, and prognostic characteristics until 1st December 2020. Additionally, the search strategy was updated on 1st January 2022. All searches were restricted to English publications. The search keywords were (“cancer” or “tumor” or “carcinoma” or “tumors” or “neoplasm” or “malignant neoplasm” or “malignant tumors”) and (“Doublecortin and CaM kinase-like-1” or “Doublecortin-like kinase 1” or “DCLK1” or “KIAA0369”). The literature search strategy is presented in Additional file 1.

### Eligibility criteria

Studies were included in this systematic review if they met the following criteria: (1) adequate data on human, in vivo, and/or in vitro CRC tumors and cells; (2) case–control and original cohort studies with CRC tumors published in English; (3) evaluated the expression of DCLK1 CSC marker by immunohistochemistry (IHC), Flowcytometry, polymerase chain reaction (RCR), reverse transcription-polymerase chain reaction (RT-PCR), or western blot (WB) in primary tumor tissues and cell lines (4) clinical studies evaluating the association between the DCLK1 expression and overall survival (OS), disease-free survival (DFS)/ relapse-free survival (RFS), and disease-specific survival (DSS)/ cancer-specific survival (CSS), and/or clinicopathological features of CRC; and (5) hazard ratios (HRs) with 95% confidence intervals (CIs) presented in the text, or availability of data in order to calculate HRs and 95% CIs.

The studies were excluded in this systematic review if they met the following criteria: (1) book chapters, reviews, letters, and conference abstracts; (2) studies were not related to the topic of the interest (e.g., when the studies investigated other solid tumors or other diseases); and (3) studies with no useful or sufficient data.

### Study selection and data extraction

All search archives were transferred to Endnote software, and duplicate records were eliminated. Two investigators (EK and MR) independently screened the titles and abstracts based on the inclusion criteria to identify the eligible studies, and discrepancies were resolved by discussion or the intervention of a third investigator (ZM). Two independent researchers (EK and FT) retrieved the data from In-vitro, In-vivo, and clinical studies in a pre-defined table. Extraction data of the In-vitro studies include the first author's name, country and year of conduction of the study, detection method, cell lines, summary of results, and signaling pathways. Extraction data of the In-vivo studies include the name of the first author, country and year of conducting the study, detection method, mouse model, summary of results, and signaling pathways. The descriptive data of clinical studies were collected for each included article: the name of the first author, country and year of conduction of the study, detection method, age, sex, sample size, median or mean follow-up times, clinicopathological parameters, cut-off value, and the related survival data. HR and 95% CI of OS, DFS/RFS, and CSS/DSS were considered for counting pooled HR. The primary outcome was the association between DCLK1 expression and OS, DFS/RFS, or CSS/DSS in CRC patients. The relationship between DCLK1 expression and important clinicopathological characteristics of CRC were also of interest. EK, MR, RG and MA.L were validated all of the extracted data.

### Quality assessment

Two independent investigators (FT and MR) assessed the quality of human clinical studies according to Newcastle–Ottawa Scale (NOS) tool [32]. NOS tool is divided into three sections: selection, comparability, and exposure or outcome, with a score ranging from 0 to 9. Based on the star of each section, the quality assessment results are classified into three groups: good, fair, and poor. Any discrepancies between the two authors were resolved by discussion or the intervention of a third investigator (ZM).

### Statistical analysis

The pooled odds ratios (ORs) with corresponding 95% CIs were applied to estimate the association between DCLK1 expression and clinicopathological characteristics. In the current analysis, an OR > 1 indicated a higher probability of tumor progression in CRC patients with high expression of DCLK1. To evaluate the effect of DCLK1 overexpression on CRC patients’ prognosis, including OS, DFS/RFS, and CSS/ DSS, the combined HRs and matching 95% CI values were calculated. Both multivariate and univariate statistical analyses were applied to extract HRs by preferring data from multivariate statistics. HRs regarding prognostic information were obtained directly from the articles if available; otherwise, we calculated by the Kaplan-Meyer curve based on the method described by Parmar et al. [33]. The software GetData Graph Digitizer (http://getdata-graph-digitizer.com/) was used to extract survival data from Kaplan-Meyer curves. The heterogeneity of the eligible publications was assessed using the I2 statistics. The random-effect framework was utilized in the presence of significant heterogeneity (p < 0.05 and/or an I2 statistic > 50%); otherwise, the fixed-effects models’ framework was applied. A combined HR greater than 1 suggested a worse prognosis in CRC patients. Funnel plots and Egger’s test were applied to graphically and statistically assess the potential publication bias, respectively. The comprehensive software Meta-Analysis (CMA Version 2.2.064) was utilized to conduct the statistical analyses. A two-tailed p < 0.05 defined statistical significance.

## Results

### Baseline study characteristics

**Fig. 2:**
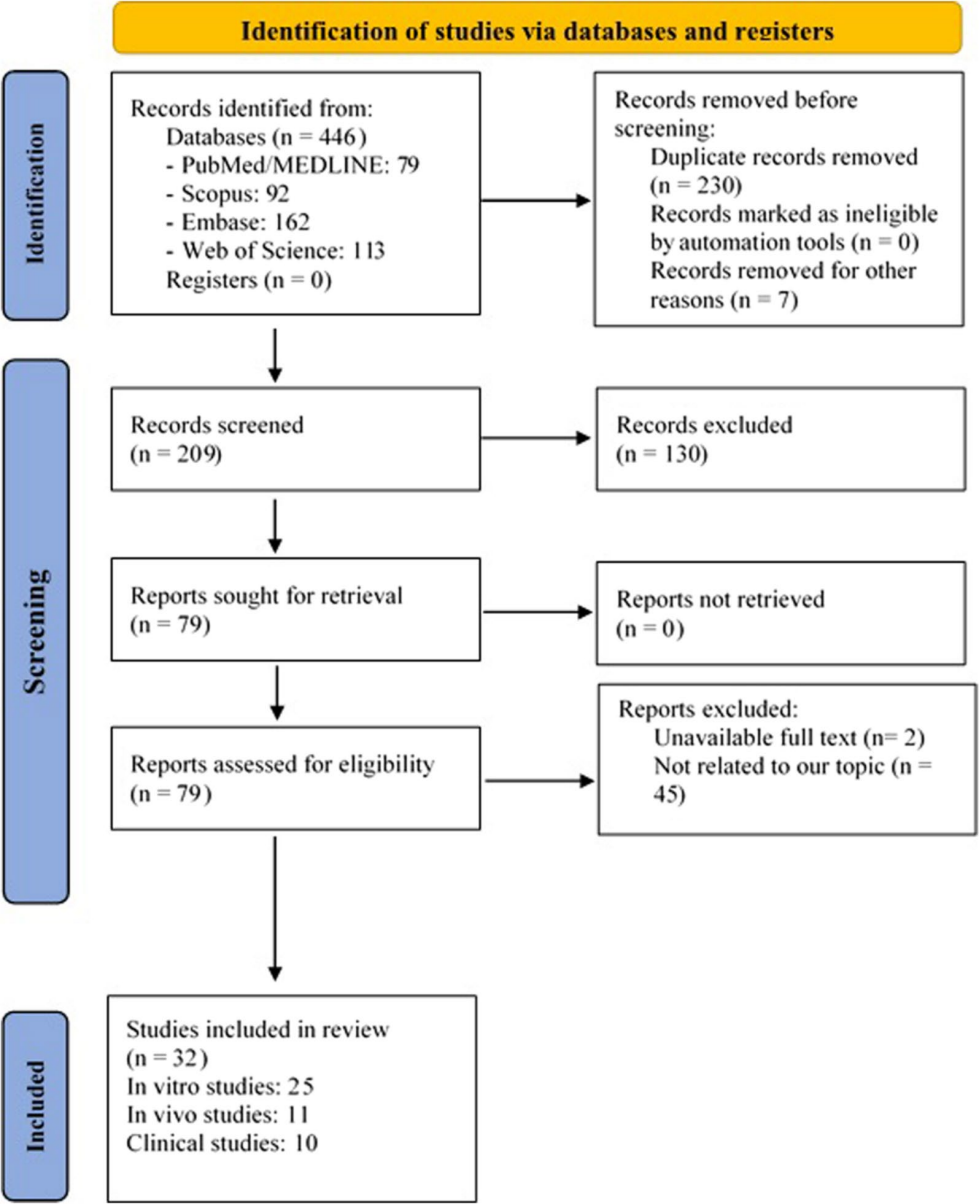
Flow chart of the search strategy according to the Preferred Reporting Items for Systematic Reviews and Meta-Analyses (PRISMA) guidelines

A literature search initially yielded 446 relevant publications from the PubMed/MEDLINE (n = 79), Scopus (n = 92), Embase (n = 162), and Web of Science (n = 113) databases until January 10, 2020 and updated on December 1, 2021. After duplicates were removed and the remaining articles reviewed, 207 records were selected. After title and abstract screening, a total of 128 records were eliminated. The full texts of the remaining 79 records were assessed for eligibility criteria, and 32 documents, including 48 studies, were included for the present systematic review and meta-analysis. Of 48 total investigations, 26 and 12 articles were in vitro and preclinical studies, respectively, and 10 studies were included in meta-analysis (Fig. 2). The essential aspects of the publications included in this study as well as the demographics of the patients are described in the clinical study section. All of the eligible articles were published in English between 2007 and 2022 and had sample sizes ranging from 71 to 348 specimens. Geographically, the majority of the studies (n = 17) were conducted in the U.S., while the rest (n = 15) were carried out in other countries (China, Iran, Japan, Korea, Norway, and Italy). For 10 tissue analysis (clinical studies), 9 articles used the IHC detection method and one used qRT-PCR to measure the expression of DCLK1. Three scoring methods, i.e. intensity of staining, percentage of positive cells, or histochemical score (*H*-score), were used to determine cut-off values. Based on the antibodies used in these publications, isoform-classification of our data was categorized in three main groups: (i) studies based on the DCLK1-L isoform using antibodies could detect the specific fragment of the DCLK1-L isoform; (ii) studies based on the DCLK1-S isoform using antibodies could detect specific sequence of DCLK1-S isoform; and (iii) studies based on using antibodies could detect the sequence homology epitopes of both DCLK1-L and DCLK1-S isoforms, known as anti-DCLK1-L/S antibodies in the current review.

**Table 1:** Summary of In vitro studies and results

Author, year, Country	Cell line	Isoform	Results
Gao, T. 2016, China	SW480	DCLK1-L/S	High expression of DCLK1 led to: Promote the migration and invasion Decrease in E cadherin expression, however enhanced the expression of Vimentin and ZEB1, which are mesenchymal markers
O'Connell, M. R. 2015, USA	HEK293 and HCT116	DCLK1-S	Identify specific functions of DCLK1 isoforms: Proposed an alternative β-promoter activator, known as NF-κBp65, that aids us in preventing colon cancer by DCLK1-S, which might be a critical target
Roy, B. C. 2019, USA	HCT116 and SW480	DCLK1-L/S	Examined the presence of autophagy: Increased DCLK1 promoter activity followed by co-localization of p62/Dclk1, which is led to inhibit DCLK1’s removal
Sarkar, S. 2017, USA	HEK293, HCT116, COLO-205, RKO, COLO-320, SW1417, CCD841	DCLK1-S	High expression of DCLK1-S resulted in decreased FOXD3 that led to increase invasive potential in cells
Sarkar, S. 2017, USA	HEK293, HCT116, COLO-205	DCLK1-S	Generating PS41014 antibody, an anti DCLK1-S antibody, which could be used to assess CRC risk
Suehiro, Y. 2018, Japan	COLO-320	DCLK1-L	Proposed combination of LRRK, a DCLK1 inhibitor, and 5-FU as a potential treatment
Vedeld, H. M. 2014, Norway	6 bile duct, 4 urinary bladder, 8 breast, 19 colon, 2 gall bladder, 4 gastric, 4 kidneys, 3 leukemia, 4 lung, 1 MPNST, 4 ovarian, 6 pancreatic, 1 prostate, 4 testis, and 4 uterus cancer cell lines	DCLK1-L	Established that hypermethylation of DCLK1 promoter could be an early detection biomarker in CRC
Venugopal, A. 2017, USA	HCT 116 and DLD-1	N/A	Promote self-renewal ability of CRC cells
Weygant, N. 2016, USA	SW480	DCLK1-L	Upregulation of DCLK1, promote EMT of CRC cells as determined
Weygant, N. 2014, USA	HCT116, HT-29, and DLD-1	DCLK1-L/S	Inhibition DCLK1 by LRRK2-IN-1 LRRK2-IN-1 inhibits proliferation DCLK1 promotes resistance to LRRK2-IN-1
Westphalen, C. B. 2014, USA	SW105 R26-Tom, R26-TGFP, or R26-DTA mice	DCLK1-L	Promote quiescence and tumor initiating in CRC cells
Makino, S. 2020 Japan	SW480, HCT116	DCLK1-L/S	Detecting high expression of DCLK1: Established the invasion and migration role of DCLK1 in CRC cells as determined DCLK1 upregulation induce EMT in CRC cells Found the positive correlation between DCLK1 and TRIB3 Found the effectiveness of combination therapy L-OHP with LRRK2
Mohammadi, Y. 2018 Iran	SW-48 and HCT-116	N/A	High expression of DCLK1 promote invasion and migration and decrease apoptosis and sphere forming ability in CRC cells
Chandrakesan, P. 2015, USA	Apc^*Min/*+^ mice IEC	N/A	Promote self-renewal ability of CRC cells
Chandrakesan, P. 2014, USA	Apc^*Min/*+^ mice IEC	N/A	Established that DCLK1 upregulation promote EMT, pluripotency, and self-renewal ability of CRC cells
Sureban, S. M. 2020, USA	HT29, HCT116, and LoVo	DCLK1-L/S	Application of DCLK1 as a molecular target in monoclonal antibody-based CAR-T cell therapy, which could be induce cytotoxicity Suggesting that high surface expression of DCLK1 is associated with increased cologenic capacity of CRC cells
Sureban, S. M. 2011, USA	HCT-116	DCLK1-L	Application of DCLK1 as a molecular target in nanoparticle (NP)-siDCAMKL-1therapy
Wu, X. 2016, China	SW480 and SW620	DCLK1-L	Establishing the combination therapy of 5-FU with XAV939 led to downregulation of DCLK1 in CRC cells
Dai, T. 2018, China	NCM460 and HCT116	DCLK1-L DCLK1-S	Isoform specific function of DCLK1 by generating monoclonal antibodies
Mohammadi, C. 2021, Iran	HCT-116	N/A	High expression of DCLK1 promote EMT and cell proliferation, decreased apoptosis
Park, S. Y. 2019, Korea	HCT116, HT29, SW480	DCLK1-L DCLK1-S	Induce cancer stemness by confining the LEF1/DCLK1 axis through niclosamide
May, R. 2009, USA	SW480	DCLK1-L	Introduce DCLK1 as a cell surface marker
Li, L. 2019, USA	HCT116, WT	N/A	DCLK1 upregulation induce chemoresistance with 5-FU treatment by inhibiting apoptosis activity
Li, L. 2013, USA	HCT116	DCLK1-L	Induce apoptosis and self-renewal ability of CRC cells by FACS, ELDA, qRT-PCR
Lu, Y. 2018, Japan	HCT116, HEK293FT, H1299, MCF10A	N/A	Dclk1 induce DNA damage assessed DCLK1 promotes cell proliferation

### Quality assessment of clinical studies

**Fig. 3:**
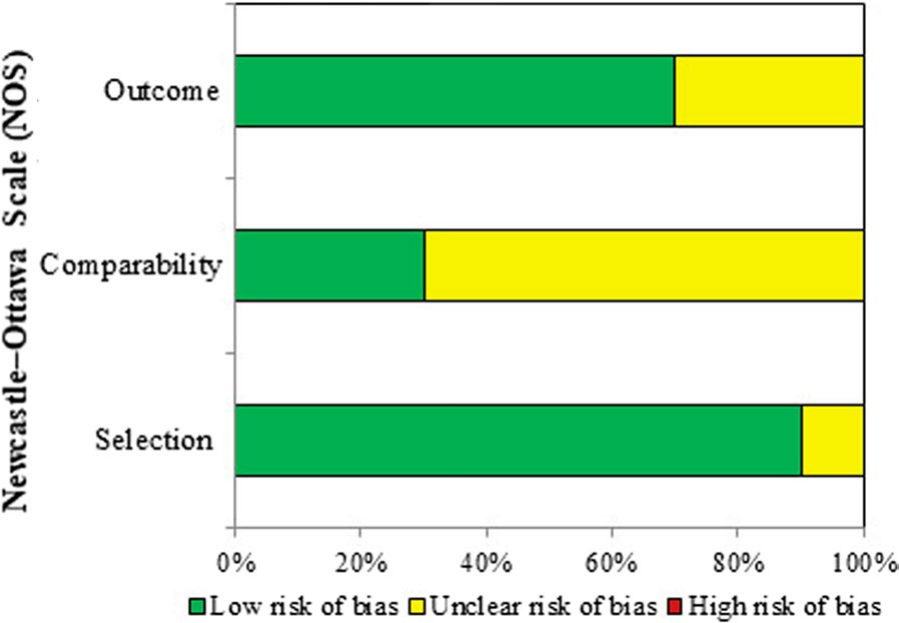
Summary of risk of bias assessment of clinical studies

Based on the NOS quality assessment, nine articles were classified as good quality and one as fair quality. The results of risk of bias assessment for each included clinical study are provided in Fig. 3.

### Findings from in vitro studies

A total of 25 studies investigated the oncogenic role of DCLK1 in vitro. The DCLK1 detection methods employed by the majority of these studies included quantitative real-time PCR, reverse transcription PCR, and Western blotting. All detailed data is collected in Table 1. A wide range of colorectal cancer cell lines was utilized, including HCT116, HT 29, SW480, COLO-320, COLO-205, DLD-1, HEK293, etc. Furthermore, one study investigated mice cell lines derived from the intestine, colon, and antrum of R26-Tom, R26-TGFP, and R26-DTA mice, respectively [20]. In addition, there is strong experimental evidence that involves DCLK1 in the acquisition of many hallmarks of cancer, including proliferation, invasion, metastasis, angiogenesis, and cell resistance. Table 1 presents an overview of the included in vitro publications that investigated the function of DCLK1 and its signaling pathways in various CRC cell lines, and the results of the studies are further explained below in detail.

Based on our isoform-specific classification, nine studies used anti-DCLK1-L antibodies, four studies used specific anti-DCLK1-S antibodies, and five studies used anti-DCLK1-L/S antibodies. Eight articles did not give detailed information regarding the used anti-DCLK1 antibodies.

DCLK1-L, originated from α-promoter, for the first time in 2013 and known as a distinguished gastrointestinal (GI) CSC marker. In multiple intestinal neoplasia (Min), mice carry a dominant mutation in the adenomatous polyposis coli (Apc) gene, called Apc^Min/+^ [11]. Therefore, the role of alternative variants of DCLK1 was highlighted. DCLK1-S is expressed from β-promoter and was recognized for the first time in 2015 as a potential CRC cancer stem cell biomarker [34]. O'Connell M.R. et al. proposed an alternative β-promoter activator, known as NF-κBp65, to aid in preventing colon cancer by DCLK1-S, which might be a critical target [34].

The oncogenic functions of DCLK1 in CRC are divided into six main categories described below:

#### Invasion

One study used specific anti-DCLK1-S antibody to review the invasion function of DCLK1. Forkhead box D3 (FOXD3) has a potential inhibitory role in invasion and progression of tumors. The study established that FOXD3 expression is downregulated in CRC cells compared to normal cells, while DCLK1-S expression is elevated. The results also highlighted the potential suppressor function of FOXD3 in the regulation of DCLK1-S in normal cells [29].

#### Epithelial-mesenchymal transition (EMT)

Based on the information in five articles, the association between epithelial-mesenchymal transition (EMT) and DCLK1 expression was analyzed [14, 17, 35–37]. EMT plays a staple role in both invasion and metastasis of cancers. DCLK1 contributes to the promotion of EMT by means of the overexpression of mesenchymal markers (Vimentin) and transcriptional regulators (ZEB1) and the downregulation of the epithelial marker E-cadherin in colon cancer cells [14, 17, 35]. Among these studies, two used anti-DCLK1-L/S [14, 35], one used anti-DCLK1-L antibodies [37], and two others did not give information on the anti-DCLK1 antibodies used [17, 36].

Gao et al. showed that high expression of DCLK1 results in migration and invasion of cancer cells through the up-regulation of the mesenchymal markers Vimentin and ZEB1 and down-regulation of the epithelial marker E-cadherin in the SW480 colorectal cell line [14]. Chandrakesan et al. offered evidence that the high expression of DCLK1 correlates with elevated levels of EMT factors, such as Snail, Slug, Vimentin, and pluripotency factors including Myc and Nanog [17]. Additionally, Makino et al. reported that the downregulation of DCLK1 expression results in decreased cell growth and invasion. Likewise, there was a direct relationship between DCLK1 and tribbles homolog 3 (TRIB3) expression; the high expression of TRIB3 brought about both E-cadherin downregulation and Vimentin upregulation (EMT-like profile) [35].

#### Apoptosis (resisting cell death)

Four main investigations were related to the area of apoptosis enhancement by DCLK1 downregulation. Of these, three studies did not report on the anti-DCLK1 antibodies used [36, 38, 39], and one study used both anti-DCLK1-L and anti-DCLK1-S antibodies [22, 40].

Similarly, Li et al. found that colorectal cancer patients whose tumors express a high level of DCLK1 are resistant to chemotherapy with 5-Fu through suppression of caspases gene expression, including casp3, casp-4, and casp-10, in the apoptosis pathway [38]. The integrated findings of two studies showed that decreasing DCLK1 expression fundamentally augmented the apoptosis cells and decreased cell proliferation by affecting cell cycle phases [36, 38]. In this regard, Mohammadi et al. described downregulation as being conducive to mitigating the sphere-forming ability of CRC cells, inducing apoptosis, and suppressing CRC cell invasion and migration by inhibiting DCLK1 by DCLK1 siRNA and regulating miR-200c [39]. Park et al. found that DCLK1-S depletion impairs cancer stemness, resulting in reduced survival potential and increased apoptosis, thus sensitizing colorectal cancer to chemoradiation [40].

#### Self-renewal

In the subsequent part, another prominent feature of DCLK1, which is self-renewal ability, will be depicted based on six studies. Two studies used anti-DCLK1-L antibodies [20, 26], but the remaining studies did not highlight anti-DCLK1 antibodies information [17, 22, 41].

Chandrakesan et al. executed three studies on tumorigenesis of the intestine by working on intestinal epithelial cell (IEC) cell lines. DCKL1 expression promotes intestinal tumorigenesis by increasing self-renewal capability and pluripotency [17, 22]. Similarly, Venugopal et al. achieved that increasing RNA binding motif-containing protein 3 (RBM3) expression basically contributes to enhancing self-renewal and spheroid formation in cells expressing DCLK1 [41]. To emphasize this issue, Li et al. mentioned that cells stemming from spheroids have high self-renewal capability [26]. Moreover, they asserted that DCLK1 expression increased in both normal oxygen and hypoxia condition in HCT 116 spheroids [26]. Westphalen et al. cast light on the fact that the vast majority of DCLK1 subset cells are named long-lived DCLK1 and play a considerable role in maintaining quiescence and initiating colon cancer [20].

#### DCLK1 as a therapeutic target

A total of seven studies presented varied features of DCLK1, which can be used as a candidate for targeted therapy [21, 35, 36, 40, 42–44]. From these studies, two used anti-DCLK1-L antibodies [42, 43], three employed anti-DCLK1-L/S antibodies [21, 35, 44], four did not discuss the used anti-DCLK1 antibodies [36], and only one used both anti-DCLK1 L and S antibodies [40].

Wu’s research confirmed that the combination therapy of 5-FU with XAV939 (inhibitor of WNT signaling pathway) is more efficient than treatment with 5-FU alone in decreasing DCLK-1 protein expression in SW480 cells. Wu also found that XAV939 reduces the CSC markers (EpCAM, TERT) and increases chemosensitivity in colon cancer cells by inhibiting the Wnt signaling pathway [42]. Another study pointed out that the effective treatment of CRC patients resistant to radiotherapy is to employ DCLK1 as a target therapy agent. DCLK1 expression increased in RR-HCT-116 (radioresistant cells HCT-116) and was reduced through DCLK1 siRNA transfection, indicating that the inhibition of DCLK1 could increase radiosensitivity in CRC cells [36]. Additionally, three studies offered that LRRK2, an inhibitor of DCLK1, can prevent DCLK1 kinase activity and has the effect of anti-cancer activity. Thus, the LRRK2 substance has the potential to be applied as an anti-tumor drug [21, 35, 43].

Roy et al. suggested that when HCT116 cells are treated with chloroquine (CQ) and lipopolysaccharide (LPS), the expression levels of LC3I/II, p62, and DCKL1 increased. They highlighted that these two factors could be used as activators of DCLK1-S promoters [44]. In addition, Park et al. acknowledged that DCLK1-S is the target of lymphoid enhancer-binding factor 1 (LEF1), which is directly associated with cancer development and maintaining CSC populations. They pointed out that niclosamide inhibits the attachment of LEF1 to the DCLK1-S promoter. Therefore, confining the LEF1/DCLK1 axis through niclosamide leads to the eradication of CSCs. This process can be an innovative treatment for CRC [40].

#### DCLK1 function in CRC detection

Two studies described the different aspects of DCLK1, which can be used as a diagnostic marker for CRC [45, 46]. One used anti-DCLK1-L and anti-DCLK1-S antibodies [45], and another employed anti-DCLK1-L antibodies [46].

Dai et al. engendered two monoclonal antibodies (mAbs) named DCLK1-42 (S) and DCLK1-87 (L) against DCLK1 using a novel chip-based immunospot array assay on a chip system, and both antibodies were able to recognize DCLK1 in the cytoplasm. They represented the expression of DCLK1-L in normal colon and DCLK1-S in human colon cancer cells. These monoclonal antibodies can be used as a tool to detect DCLK1 in CRC [45]. Vedeld et al. showed a negative correlation between the presence of DCLK1 promoter methylation and expression levels of the transcript. They suggested that promoter hypermethylation of DCLK1 can be an early detection biomarker in CRC [46]. Moreover, the role of DCLK1 in determining CRC risk was discussed in one research that worked on anti-DCLK1-S antibodies [30]. Sarkar et al. discovered that DCLK1-S, which was detected explicitly by the PS41014 antibody, can be employed as a target to measure CRC risk [30].

### Findings from in vivo studies

Among eleven reports on in vivo studies, three studies highlighted the oncogenic and therapeutic function of DCLK1 using DCLK1-L antibodies [20, 47, 48], five used anti-DCLK1-L/S antibodies [11, 44, 49–51], and only one study used specific anti-DCLK1-S antibodies [34] in CRC. Three articles did not give detailed information of the anti-DCLK1 antibodies used [17, 18, 22]. Table 2 presents the summarized in-vivo data. These findings demonstrated the main oncogenic functions of DCLK1 in CRC.

**Table 2:** Summery of In vivo studies and results

Author, year, Country	Mouse model	Isoform	Results
May, R. 2008, USA	C57 Bl/6	DCLK1-L	Induce tumor proliferation
May, R. 2009, USA	C57 Bl/6	DCLK1-L	Promote quiescent and increase survival
Nakanishi, Y. 2012, Japan	APC ^Min+^	DCLK1-L/S	Introduce DCLK1 as a distinguished CRC-CSC marker and a tumor initiation factor
Roy B.C. 2019, USA	APC^++^	DCLK1-L/S	Promote tumor initiation and development
Westphalen, C. B. 2014, USA	BAC-CreERT–dependent genetic lineage–tracing strategy	DCLK1-L	Induces tumor initiation
Chandrakesan, P. 2015, USA	DCLK1- CreER;Rosa26-YFP	N/A	Induced tumorigenesis, pro-survival, and quiescence
Chandrakesan, P. 2014, USA	APC ^Min/+^	N/A	Induced tumorigenesis, pro-survival, and quiescence
Femia, A. P. 2013, Italy	F344 rats	DCLK1-L/S	More neoplastic function of LGR-5 in comparison with MSA-1 and DCLK1 in normal colon mucosa, precancerous lesions, and adenoma tumors
O’Connell, M. R. 2015, USA	Friend Virus B NIH (FVB/N)	DCLK1-S	Important function of DCLK1-S in tumor proliferation and metastasis compared with DCLK1-L
Sureban, S. M. 2011, USA nano	Athymic nude mice (NCr-nu/nu)	DCLK1-L/S	Thargeted-therapy by Nanoparticle delivery
Sureban, S. M. 2020, USA cart	NOD Scid gamma (NSG)	DCLK1-L/S	Thargeted-therapy by CAR-T cell therapy

#### Tumor initiation

Westphalen et al. generated Dclk1-CreERT BAC transgenic APC^Min/+^ mice and confirmed that the small proportion of DCLK1 + cells in intestinal tuft cells had potential tumor initiation and quiescent features upon activation of pathological Wnt signaling combined with an inflammatory stimulus [20]. A valuable study of APC ^Min/ +^ mice by Nakanishi et al. revealed that DCLK1 as a colorectal CSC marker distinguishes this population from normal stem cells by lineage-tracing experiments and suggested DCLK1 as a promising therapeutic candidate in colorectal cancer [11]. Roy et al. investigated the co-expression of DCLK1 with Beclin-1, LC3B and p62 as an autophagy marker and a critical oncogenic regulator in various signaling pathways in APC ^Min/ +^ mice; indicating the oncogenic function of these markers in advanced tumorigenesis and tumor progression in colorectal cancer [44]. In contrast, Femia et al. in a comparative investigation analyzed the expression patterns of three CCSC markers, leucine-rich-repeat-containing LGR-5, MSI-1, and DCAMKL-1. They reported LGR-5 as a putative neoplastic stem cell marker in comparison with MSA-1 and DCLK1 in normal colon mucosa, precancerous lesions, and adenoma tumors [49].

#### Tumor proliferation

May et al. investigated the effect of DNA-damaging by phosphor-H2AX staining, apoptosis agents (including p53 and Musashi-1 (MSI-1)), and proliferation agents (including proliferating cell nuclear antigen (PCNA)) on the eradication of DCLK1-expressed cells in APC^min/+^ mice, and they suggested DCLK1 as a potential therapeutic target in CRC and intestinal cancer treatments [47].

#### Quiescent and increase survival

In another study, May et al. represented the gene expression profile and molecular signature of DCLK1 as a quiescent intestinal stem cell marker to increase survival of tumor stem cells (TSCs) [48]. By using NP-siDCAMKL-1, the growth of both tumor cells and proto-oncogenes (c-Myc and Notch-1) was reduced [51].

#### Tumor invasion and metastasis

OʹConnell et al. demonstrated that epigenetic changes regarding the hypermethylation of the α-promoter of DCLK1 throughout colon carcinogenesis did not happen in mouse, unlike human genes. They represented the high expression of DCLK1-L at the early stage and loss of DCLK1-S expression in CRC without metastatic lesions in Friend Virus B NIH (FVB/N) mice. Interestingly, they induced liver metastatic lesion by implanting highly metastatic HEKmGAS cells that overexpress DCLK1-S in a mouse model, indicating the important role of DCLK1-S in tumor invasion and metastasis in mouse colon tumors [34].

In three comprehensive studies, Chandrakesan et al. reported great tumorigenesis, pluripotency, quiescence, self-renewal, and EMT characteristics in DCLK1 + cells through the expression of Cdkn1A, Cdkn1B, Oct4, Sox2, Nanog, Klf4, Wif1, RelA, Akt and AMPK, and the critical regulation of Raptor, Rictor, p53, and Survivin in APC mutant mice in both mRNA and protein levels. They also verified these findings by silencing DCLK1, indicating a significant decrease in the expression of tumorigenesis, pro-survival, and quiescence in the mentioned factors [17, 18, 22].

#### DCLK1 as a therapeutic target

Sureban investigated two innovative treatments in two distinct studies for colorectal cancer patients. One research represented a novel chimeric antigen receptor (CAR-T) targeting DCLK1 as a treatment strategy to eradicate CRC CSC in HT29, HCT116, and LoVo cell lines. DCLK1-targeted monoclonal antibody (CBT-15 mAb) was used to generate CAR-T targeting DCLK1 named CBT-511. Sureban found significant cytotoxicity induction and higher interferon gamma (IFN-γ) secretion in CRC cells by CBT-511 in comparison to mock CAR-T. They also indicated that CBT-511 treatment resulted in a 50% reduction in tumor growth compared to mock CAR-T [50]. In another study, they targeted DCLK1 as a critical oncogenic regulatory factor using nanoparticle (NP) technology to deliver DCLK1-specific small interfering RNA (siRNA) in HCT116 xenografts aimed to upregulate tumor suppressor microRNAs (let-7a, miR-200a, and miR-144) for cancer therapy in CRC [51].

### Findings from clinical studies

Table 3 presents the main features of the included clinical studies and patient demographics. Notably, all the eligible articles were written in English, published between 2012 and 2022, and had sample sizes ranging from 71 to 235 participants. According to the NOS quality assessment listed in Table 3, all 10 publications were categorized as high quality with scores ranging from 6 to 9. Geographically, most of the studies (n = 8) were conducted in Asia, but two articles were performed in the U.S. Moreover, many of the participants in the majority of studies were male. Most articles (n = 9) applied the IHC detection method for analyzing tissue. Among the 10 clinical studies, 8 experiments reported the cytoplasmic localization of DCLK1-L and DCLK1-L/S expression, and one study detected DCLK1-S in the cytoplasmic area of tumor cells [52] by immunohistochemical assay. Another study detected the cytoplasmic, membranous, and nucleus expression of DCLK1-S by immune-electron-microscopy (IEM) [30]. Three studies investigated using anti-DCLK1-L antibodies, two other studies used anti-DCLK1-S antibodies, and the remaining five studied anti-DCLK1-L/S antibodies. Furthermore, five studies evaluated the prognostic value of the CSC markers on OS, while five and four investigations assessed the prognostic importance of the markers on DFS/RFS/PFS and DSS/CSS, respectively.

**Table 3:** Summary of clinicopathological characteristics of the clinical studies of CRC patients included in the meta-analysis

Authors, year (Country)	Method	Isoform	Cut-of value	Localization	Sample size (n)	Age Mean/med	Gender (M/F)	TNM stage	Outcome	NOS score#
Gao, T. 2016 (China)	IHC	DCLK1-L/S	H-score	Cytoplasmic	71	60	44/27	I–IV	CSS	8
Harada, Y. 2018 (Japan)	IHC	DCLK1-L /S	H-score	Cytoplasmic	36	62.4		I–III	CSS/RFS	7
Makino, S. 2020 (Japan)	IHC	DCLK1-L /S	H-Score	Cytoplasmic	180	61	120/60	0–IV	OS/RFS	7
Dai, T. 2018 (China)	IHC	DCLK1-L	H-score	Cytoplasmic	100	65	53/44	I–IV	N/A	6
Ikezono, Y. 2015 (Japan)	IHC	DCLK1-L	H-Score	Cytoplasmic	18	51	9/9	N/A	N/A	6
Sarkar, S. 2017 (USA)	qRT-PCR	DCLK1-S	Image	Cytoplasmic/membranous/nucleous	92	67	57/35	I–IV	OS/ DFS	7
Gagliardi, G. 2012 (USA)	IHC	DCLK1-L	H-score	Cytoplasmic	71	58	38/20	I–IV	CSS	9
Razi, S. 2020 (Iran)	IHC	DCLK1-L/S	H-score	Cytoplasmic	181	60	87/93	I–IV	N/A	7
Kalantari, E. 2021 (Iran)	IHC	DCLK1-S	H-score	Cytoplasmic	235	60	177/165	N/A	DSS/PFS	7
Kang, X. 2020 (China)	IHC	DCLK1-L/S	H-score	Cell membrane	92	49	52/40	II–III	OS/PFS	6

#### DCLK1 expression and its association with survival outcomes in CRC patients

**Fig.4:**
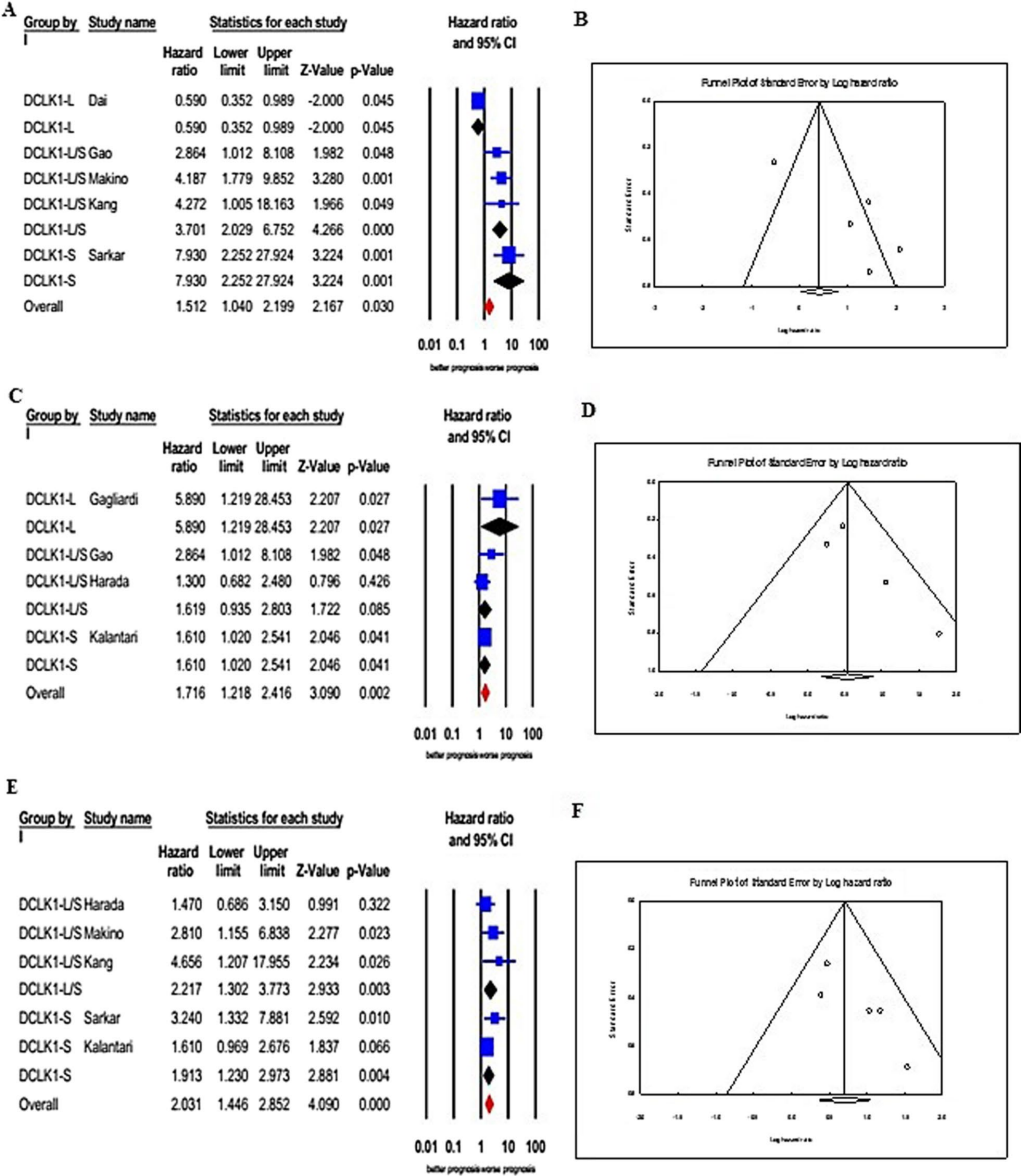
Forest (**A**, **C**, and **E**) and Funnel (**B**, **D**, and **F**) plots for the association of DCLK1 overexpression with survival. A and B DCLK1 overexpression association with overall survival (OS); **C** and **D** DCLK1 overexpression association with disease/cancer-specific survival (DSS/CSS); **E** and **F** DCLK1 overexpression association with disease/relapse/progression-free survival (DFS/RFS/PFS)

Statistical results of survival outcomes are shown in the forest plots in Fig. 4. Five studies involving 535 patients evaluated the relationship between DCLK1 expression and OS. Because of the significant heterogeneity between studies, the random effects framework was adopted to combine the HRs (I2 = 85.842%, *p* < 0.001). The results disclosed that DCLK1 overexpression was associated with inferior OS (pooled HR = 1.512, 95% CI 1.040–2.199, *p* = 0.030). However, relatively significant publication bias was identified in either the funnel plot (Fig. 4) or Egger’s test (*p* = 0.0388). When the investigations were stratified based on different DCLK1 isoforms, a poor OS was observed for three studies with DCLK1-L/S (HR = 3.701, 95% CI 2.029–6.752, *p* < 0.001) and one study with DCLK1-S (HR = 7.930, 95% CI 2.252–27.924, *p* = 0.001), while increased expression of DCLK1-L (n = 1) was closely associated with better OS in CRC patients (HR = 0.590, 95% CI 0.352–0.989, *p* = 0.045).

Four studies evaluated the impact of DCLK1 overexpression on DSS/CSS in a total of 563 CRC patients. As represented in Fig. 4, a fixed-effect framework was employed owing to limited heterogeneity among the studies (I2 = 26.308%, *p* = 0.254). The pooled HR exhibited that the overexpression group of DCLK1 displayed a statistically considerable decrease in DSS/CSS (pooled HR = 1.716, 95% CI 1.218–2.416, *p* = 0.002). Both the funnel plot (Fig. 4) and Egger’s test (*p* = 0.155) revealed that there was no significant publication bias. As for different DCLK1isoforms, increased expression of DCLK1-L (HR = 5.890, 95% CI 1.219–28.453, *p* = 0.027) was closely associated with worse DSS/CSS in CRC patients compared to DCLK1-S (HR = 1.610, 95% CI 1.020–2.541, *p* = 0.041).

Five studies comprising 785 cases evaluated the prognostic role of DCLK1 on cancer progression or recurrence. No significant heterogeneity existed across the studies (I2 = 11.459%, *p* = 0.340). Under a fixed-effect framework, the pooled results demonstrated that elevated expression of DCLK1 predicted worse DFS/RFS/PFS in patients with CRC in comparison to low DCLK1 expression with a combined HR of 2.031 (95% CI 1.446–2.852, *p* < 0.001, Fig. 4). No significant publication bias was observed in either the funnel plot (Fig. 4) or Egger’s test (*p* = 0.074). Stratified analysis by different DCLK1 isoforms indicated a poor DFS/RFS/PFS for three studies with DCLK1-L/S (HR = 2.217, 95% CI 1.302–3.773, *p* = 0.003) and two studies with DCLK1-S (HR = 1.913, 95% CI 1.230–2.973, *p* = 0.004). However, no study on the association between DCLK1-L and DFS/RFS/PFS in CRC patients was found.

#### DCLK1 expression and its association with clinicopathological features in CRC patients

**Fig.5:**
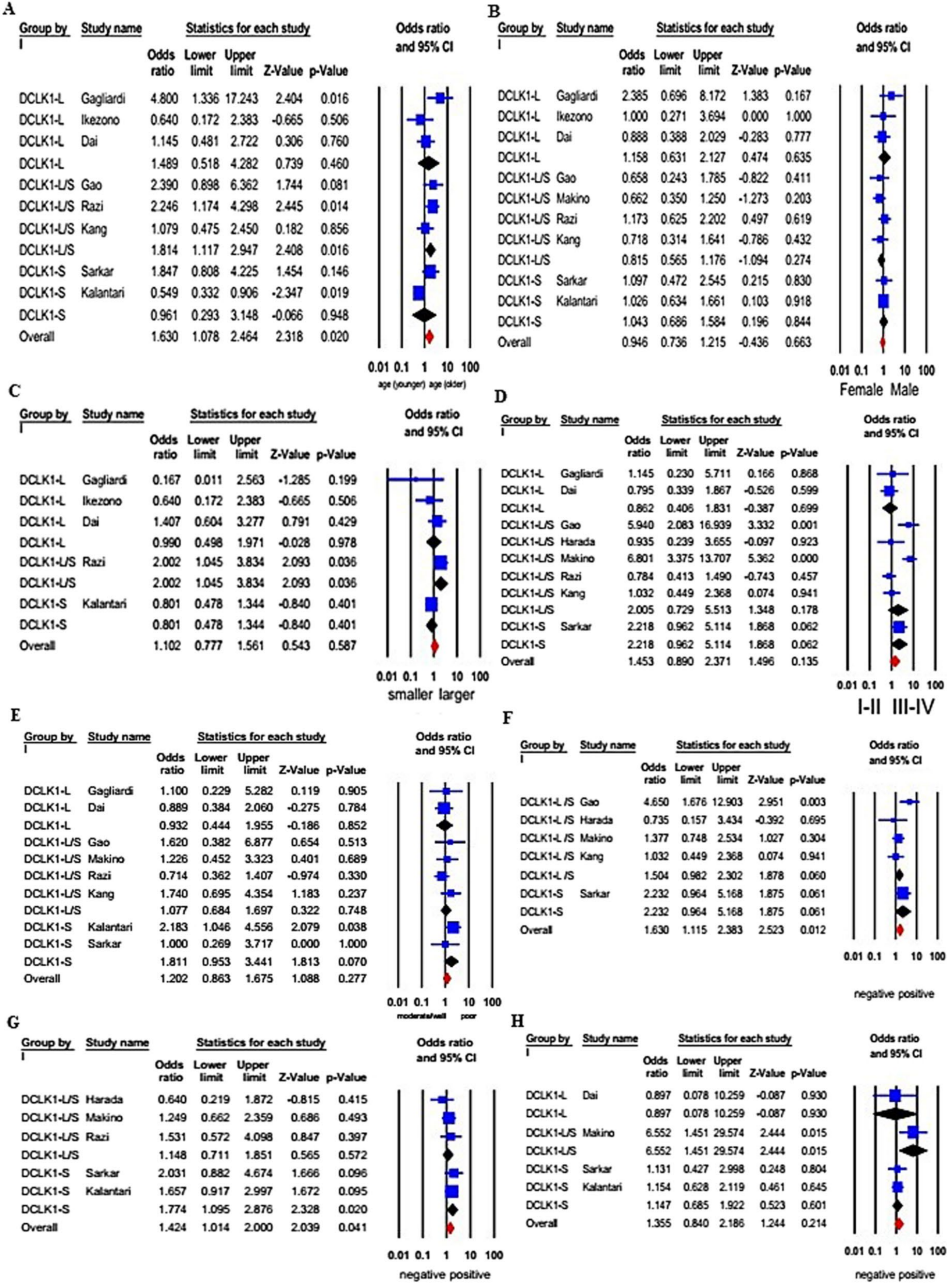
Forests plots for the association between DCLK1S/L expression and clinicopathological parameters. **A** age; **B** Gender; **C** tumor size; **D** TNM stage; **E** differentiation; **F** lymph node metastasis (LNM); **G** vascular invasion; **H** distant metastasis

A total of eight studies with 955 patients examined the association between DCLK1 expression and age. A random-effect framework was applied, because significant heterogeneity was identified (I^2^ = 67.239%, *p* = 0.003). The combined results clarified that DCLK1 was preferably expressed in patients with older ages (pooled OR = 1.630, 95% CI 1.078–2.464, *p* = 0.020; Fig. 5). These results indicated that DCLK1 was closely associated with an older age for the onset of CRC. Stratified analysis based on different isoforms indicated that only studies with DCLK1-L/S (n = 3) showed an association with older ages (OR = 1.814, 95% CI 1.117–2.947, *p* = 0.016).

In total, five studies comprising 673 patients revealed a connection between DCLK1 expression and tumor size. A fixed-effect model was adopted due to insignificant heterogeneity (I^2^ = 46.750%, *p* = 0.111). Obviously, this association demonstrated that patients with increased DCLK1 expression were not liable to develop large-sized tumors (OR = 1.102, 95% CI 0.777–1.561, *p* = 0.587, Fig. 5). As for different DCLK1 isoforms, only increased expression of DCLK1-L/S (n = 1) was closely associated with large tumor size (HR = 2.002, 95% CI 1.045–3.834, *p* = 0.036).

The relationship between DCLK1 expression and lymph node metastasis was evaluated in five studies including 461 patients. A fixed-effect framework was employed to estimate the combined OR and matching 95% CI as there was insignificant heterogeneity between studies (I^2^ = 43.468%, *p* = 0.132). The pooled results suggested that cases with overexpression of DCLK1 preferentially metastasized to the lymph nodes (OR = 1.630, 95% CI 1.115–2.383, *p* = 0.012; Fig. 5). However, no significant association was found between increased expression of either DCLK1-L/S (n = 4) or DCLK1-S (n = 1) and positive lymph node metastasis.

A pooled analysis of five studies with 805 patients confirmed that DCLK1 expression was associated with vascular invasion (OR = 1.424, 95% CI 1.014–2.000, *p* = 0.041; I^2^ = 0.000%, *p* = 0.515; Fig. 5). Subgroup analyses stratified by different DCLK1 isoforms revealed that only high DCLK1-S expression was significantly associated with vascular invasion (OR = 1.774, 95% CI 1.095–2.876, *p* = 0.020).

Five studies comprising 605 patients investigated the relationship between DCLK1 expression and distant metastasis. A fixed-effect model was employed because of insignificant heterogeneity (I^2^ = 36.310%, *p* = 0.194). Pooled results indicated that cases with elevated DCLK1 expression were less likely to metastasize to distant sites (OR = 1.355, 95% CI 0.840–2.186, *p* = 0.214, Fig. 5). As for different DCLK1 isoforms, only increased expression of DCLK1-L/S (n = 1) was closely associated with distant metastasis (OR = 6.552, 95% CI 1.451–29.574, *p* = 0.015).

The relationships between augmented expression of DCLK1 and gender, differentiation, and TNM stage were also assessed. No statistically significant link was identified between DCLK1 expression, overall or individually, and the mentioned clinicopathological features (Fig. 5).

## Discussion

To date, several reviews have been conducted regarding the importance of DCLK1 in cancer initiation and progression [20, 22, 26, 53]; however, data from the available literature does not provide a complete overview on each DCLK1 isoforms, DCLK1-L and DCLK1-S. Therefore, based on the importance of DCLK1 as a specific CRC CSC marker, this comprehensive systematic review and meta-analysis was aimed to assess the oncogenic isoform-specific functions of DCLK1-L and DCLK1-S in in-vitro and in-vivo evidence and to evaluate the prognostic significance of DCLK1 isoforms in colorectal cancer. Importantly, the expression level of DCLK1 was detected by IHC and RT-PCR methods in the articles which used anti-DCLK1-L antibodies that detect N-terminal end of DCLK1-L isoform, anti-DCLK1-S antibodies that detect the specific N-terminal end of DCLK1-S isoform, and anti-DCLK1-L/S antibodies that detect similar sequences at the C-terminal end of both DCLK1-L and DCLK1-S isoforms. By including 785 CRC patients in this meta-analysis, the findings provide evidence for the significant association between DCLK1 expression and CRC prognosis, indicating the potential clinical utility of DCLK1 in CRC, although the specific difference in DCLK1 isoforms expression (DCLK1-L and DCLK1-S) in CRC has been ignored in these studies. It must be emphasized that study heterogeneity and publication bias were also addressed herein.

Although the clinical findings revealed a robust association between a higher expression level of DCLK1 and poor prognostic significance of CRC, the restricted number of studies included in this meta-analysis was a major limitation. The increased expression of DCLK1-L/S was significantly related to older age, larger tumor size, and positive distant metastasis, while higher expression of DCLK1-S was significantly associated with positive vascular invasion. Furthermore, based on different DCLK1 isoforms, high expression levels of DCLK1-S were significantly correlated with poor OS, DSS/CSS, and DFS/RFS/PFS compared to low DCLK1-S expression. There was a significant association between high DCLK1-L/S expression and worse OS and DFS/RFS/PFS, while increased expression of DCLK1-L was significantly associated with better OS in CRC patients. Additionally, majority of the survival analyses was lack of publication bias in the pooled HR for either survival analysis or overall DCLK1 expression. The clinicopathological and survival outcomes of the current meta-analysis represented a similar prognostic significance pattern for DCLK1-S and DCLK1-L/S and may emphasize their critical roles in CRC prognosis and patient surveillance, possibly because DCLK1-S is the predominant isoform in CRC tissue. Nonetheless, further studies are required to show different expressions of these two isoforms. These findings suggest that DCLK1-S (as opposed to DCLK1-L isoform) can be a potential CSC marker as a therapeutic marker in CRC to increase the survival rate of these patients. In line with other studies, no significant association was observed between DCLK1 expression and tumor differentiation in CRC patients [14, 54]. According to the current findings, DCLK1-S plays a key role in the relapse of CRC (HR = 2.124) compared to death resulting from CRC (HR = 1.849). The limited sample size for survival statistical analysis was a drawback of this systematic review; therefore, the findings should be considered with caution. Furthermore, the heterogeneity of clinical outcomes of DCLK1 expression and various cut-off thresholds used to demonstrate the positive staining of DCLK1 expression counted as other identified limitations. Another limitation of the current study was the lack of evidence on the tissue expression patterns and prognostic significance of DCLK1-S and DCLK1-L in CRC human tissues by IHC concurrently.

Preclinical and in vitro evidence have suggested the potential oncogenic roles of DCLK1 in tumorigenesis, tumor maintenance, invasion, migration, apoptosis, metastasis, and drug resistance in CRC [9, 19, 27, 39, 47, 48, 53, 55].

Nakanishi et al. reported DCLK1 as a specific colorectal CSC marker that clearly distinguishes CSCs from normal stem cells (NSCs) [11]. However, the antibody used in their study could detect the similar sequences of both DCLK1-L and DCLK1-S isoforms. Epigenetic evidence revealed that the hypermethylation of 5ʹ α-promoter in colon carcinogenesis led to loss of DCLK1-L expression in cancerous cells, and alternative β-promoter activity in response to signaling pathways caused the expression of DCLK1-S in cancer cells [56]. Among 26 in vitro studies on a wide range of CRC cell lines, four focused on DCLK1-S using mono-specific anti-DCLK1-S antibodies. The positive expression of DCLK1-S has been detected in 80–90% of a wide range of CRC cell lines; however, DCLK1-L expression was not found in these cells by either western blot or RT-PCR analysis [34, 45]. Furthermore, the positive expression of DCLK1-L was found in normal and precancerous CRC tissues and cells [30, 34].

While most of the literature reported the biological functions of DCLK1 related to inducing EMT, self-renewal, sphere forming, maintaining quiescence, initiating colon cancer, and resisting cell death in CRC cell lines (13, 15, 17, 40, 57), it is worth mentioning that the anti-DCLK1 antibodies generally utilized in these studies could detect sequence homology epitopes of both isoforms (anti-DCLK1 L/S); hence, the specific implication of each DCLK1 isoform requires further investigation. Recently, other studies have revealed that cancer cells with positive DCLK1-S expression have more effective oncogenic activities in tumor invasion and metastasis compared to DCLK1-L positive cells in human CRC versus normal colon cell lines [12, 30, 56]; however, it seems that more definite explorations are still needed.

In vivo studies have confirmed that DCLK1 plays an oncogenic role in tumor initiation, proliferation, and quiescence due to activation of Wnt and Notch1 signaling pathways in addition to stimulating inflammation in mouse models of colon cancer [12, 16, 18, 20, 22, 44]; the antibodies used in these experiments could recognize the similar epitopes of both DCLK1-L and DCLK1-S isoforms. Thus, the isoform-specific roles of DCLK1 are poorly understood. The evidence based on DCLK1-S antibodies has highlighted the significant differences in expression patterns of the DCLK1 isoforms, DCLK1-L and DCLK1-S, in both mouse and human, indicating the major oncogenic application of DCLK1-S in tumor growth and metastasis in human [34]. Because of the lack of evidence on distant metastasis in mice models of CRC and the negative expression of DCLK1-S by in vivo experiments, it is, therefore, concluded that the absence of DCLK1-S expression may cause no tumor aggressiveness and metastasis in mouse models [34, 56]. Although using in vivo discoveries can shed new light on early detection and/or targeted-therapy strategies of human cancers, significant epigenetic changes of DCLK1 isoforms expression in mice and humans highlight the requisite advances to generate mice models with fewer micro-environment differences and more epigenetic similarities to human CRC tumors.

## Conclusion

In conclusion, the findings of the current comprehensive systematic review and meta-analysis represent the notable roles of DCLK1 CSC markers in cancer progression and predicting poor clinical outcomes of CRC patients. To the best of our knowledge, this is the first review to systematically assess the in vitro and in vivo findings of DCLK1 studies with a focus on isoform-specific functions as well as prognostic values of each isoform, including DCLK1-S and DCLK1-L, among colorectal cancer patients, which makes the findings more robust. Our preclinical findings represented the different oncogenic roles of DCLK1-L and DCLK1-S isoforms, specifically short isoforms in tumor initiation, growth, and invasion, by in vitro and in vivo evidence. However, some investigations did not specify the isoform-specific function of DCLK1, and some used anti-DCLK1-L/S antibodies which could recognize the similar sequences of DCLK1-L and DCLK1-S isoforms. The current clinical findings demonstrated dominant cytoplasmic and partially membranous localization of DCLK1-L in the tumor area, while DCLK1-S was detected in cytoplasmic, membranous, and nucleus localization in the tumor area. Furthermore, the meta-analysis results highlighted that DCLK1-S plays a critical role in the aggressive behavior of tumor cells and counts as a poor prognostic marker related to OS, DSS/CSS, and DFS/RFS/PFS in CRC. However, further investigations on the potential of utilizing DCLK1-S as a specific CRC-CSC marker to prediction and/or treatment of CRC patients are required for novel targeted-therapy strategies.

## Supplementary Information

**Additional file 1.** Search strategy of electronic databases.

## Data Availability

Not applicable.

## Abbreviations

CRC: Colorectal cancer
CSC: Cancer stem cell
DCLK1: Doublecortin-like kinase 1
DCLK1-L: DCLK1 long isoform
DCLK1-S: DCLK1 short isoform
HRs: Hazard ratios
CIs: Confidence intervals
ORs: Odds ratios
EMT: Epithelial-mesenchymal transition
OS: Overall survival
DFS: Disease-free survival
PFS: Progression-free survival
RFS: Relapse-free survival
DSS: Disease-specific survival
CSS: Cancer-specific survival
Lgr5: Leucine-rich repeat-containing G-proteinecoupled receptor 5
ALDH1: Aldehyde dehydrogenase isoform 1
GI: Gastrointestinal
Apc: Adenomatous polyposis coli
RCR: Polymerase chain reaction, RT-PCR: Reverse transcription polymerase chain reaction, WB: Western blot
IEC: Intestinal epithelial cell
RBM3: RNA binding motif-containing protein 3
CQ: Chloroquine
LPS: Lipopolysaccharide
LEF1: Lymphoid enhancer-binding factor 1
mAbs: Monoclonal antibodies
MSI-1: Musashi-1
FOXD3: Forkhead-Box-D3
TSCs: Tumor stem cells
NSCs: Normal stem cells
CAR-T: Chimeric antigen receptor
IFN-γ: Interferon-gamma
siRNA: Specific small interfering RNA
IEM: Immune electron microscopy
IHC: Immunohistochemistry
H-score: Histochemical score
