# Supplementary material for: Oncogenic functions and clinical significances of DCLK1 isoforms in colorectal cancer: a systematic review and meta-analysis
Source: Cancer Cell Int. 2022 Jun 18;22:217. doi: 10.1186/s12935-022-02632-9 (PMC9206744; doi:10.1186/s12935-022-02632-9)
Supplement: Supplementary file 1 — Additional file 1. Search strategy of electronic databases. [file 12935_2022_2632_MOESM1_ESM.pdf]

WWW.NativeEnglishEdit.com

EDITORIAL CERTIFICATE LETTER

This document is to certify that the manuscript listed below was edited for proper English language, grammar, punctuation, spelling, and overall style by one of the highly qualified subject-expert native English speaking editors at **NativeEnglishEdit.com**

The substantive content of the article mentioned below remains the full responsibility of the author/authors:

TITLE OF ARTICLE:
ONCOGENIC FUNCTIONS AND CLINICAL SIGNIFICANCES OF DCLK1 ISOFORMS IN COLORECTAL CANCER: A SYSTEMATIC REVIEW AND META-ANALYSIS

AUTHOR(S):

ELHAM KALANTARI, MAHDIEH RAZMI, FATEMEH TAJIK, MOHSEN ASADI-LARI, ROYA GHODS, ZAHRA MADJD

REFER CODE:
EE-2022-77776703-KALANTARI-5291-MED

Native English Edit
www.NativeEnglishEdit.com

Documents receiving this certification should be English-ready for publication; however, the author has the ability to accept or reject our suggestions and changes.
This certificate may be verified at:
www.NativeEnglishEdit.com
London
East End Road 27, N 3 3QT
United Kingdom
